# Prenatal Diagnosis of a 2.5 Mb De Novo 17q24.1q24.2 Deletion Encompassing* KPNA2* and* PSMD12* Genes in a Fetus with Craniofacial Dysmorphism, Equinovarus Feet, and Syndactyly

**DOI:** 10.1155/2017/7803136

**Published:** 2017-03-29

**Authors:** Marie-Emmanuelle Naud, Lucie Tosca, Jelena Martinovic, Julien Saada, Corinne Métay, Loïc Drévillon, Virginie Benoit, Sophie Brisset, Gérard Tachdjian

**Affiliations:** ^1^AP-HP, Service d'Histologie, Embryologie et Cytogénétique, Hôpitaux Universitaires Paris Sud, Site Antoine Béclère, Clamart, France; ^2^Faculté de Médecine Paris Sud, Université Paris Sud, Le Kremlin Bicêtre, France; ^3^AP-HP, Unité de Fœtopathologie, Hôpitaux Universitaires Paris Sud, Site Antoine Béclère, Clamart, France; ^4^AP-HP, Service de Gynécologie Obstétrique, Hôpitaux Universitaires Paris Sud, Site Antoine Béclère, Clamart, France

## Abstract

Interstitial 17q24.1 or 17q24.2 deletions were reported after conventional cytogenetic analysis or chromosomal microarray analysis in patients presenting intellectual disability, facial dysmorphism, and/or malformations. We report on a fetus with craniofacial dysmorphism, talipes equinovarus, and syndactyly associated with a de novo 2.5 Mb 17q24.1q24.2 deletion. Among the deleted genes,* KPNA2 *and* PSMD12 *are discussed for the correlation with the fetal phenotype. This is the first case of prenatal diagnosis of 17q24.1q24.2 deletion.

## 1. Introduction

Interstitial 17q24.1 or 17q24.2 deletions were reported in patients with malformations, facial dysmorphism, and intellectual disability [1–8]. First cases of 17q24.1 and/or 17q24.2 deletion were described by conventional cytogenetic techniques only [1–3]. Among the observed malformations, the newborn described by Levin's study had a hypertelorism associated with too large ocular globes, small and low-set ears, 2-3 toes' syndactyly, and hallux deviated by extreme flexion [1]. Conventional cytogenetic analysis revealed a 17q23.2q24.3 deletion. Clubfoot was observed in two patients [2, 3]. One case was associated with polyhydramnios at ultrasound examination during pregnancy [3]. Use of microarray comparative genomic hybridization (array-CGH) allowed the diagnosis of interstitial 17q24.1 or 17q24.2 deletions in patient suffering from intellectual disability, abnormality of the facial shape, and/or malformations [4–8]. A new syndromic entity associated with 17q24.2 microdeletion was recently described including intellectual disability, speech delay, truncal obesity, and craniofacial dysmorphism [5]. We report the first case of prenatal diagnosis of a 2.5 Mb de novo 17q24.1q24.2 deletion, by using array-CGH in a fetus with abnormal facial shape (retrognathia), talipes equinovarus, syndactyly, and mild polyhydramnios.

## 2. Clinical Presentation

A 27-year-old primigravida woman was referred to our prenatal diagnosis center at 25 weeks of gestation (WG) for fetal retrognathia, talipes equinovarus, and mild polyhydramnios. The couple was nonconsanguineous and the mother had history of clubfeet at birth. After confirmation of moderated fetal retrognathia, talipes equinovarus, and mild polyhydramnios, informed consent for genetic analysis was signed by the parents (according to the local ethical guidelines) and amniotic fluid was sampled at 25 WG.

**Figure 1:**
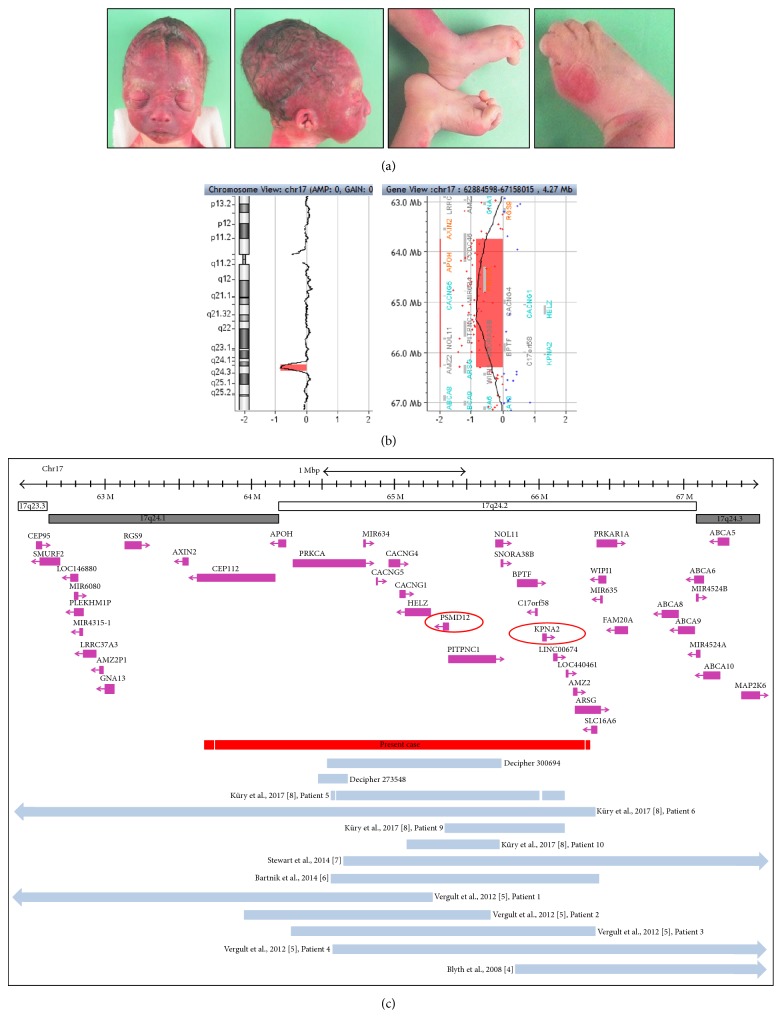
Fetal phenotype at 30 WG, fetal cytogenetic assays, and profile of the overlapping deletions described by array-CGH. (a) Fetal phenotype: face; right profile global view of the feet showing equinovarus feet and extreme flexion of the hallux; right foot with 2-3 toes' cutaneous syndactyly. (b) Agilent PreCytoNem 105K array-CGH profile of chromosome 17 showing a 2.5 Mb 17q24.1q24.2 deletion. (c) Profile of the overlapping deletions described by array-CGH (patients of Blyth et al. and Stewart et al. were remapped in hg19).

After genetic counseling and according to the French Law, the pregnancy was terminated at 30 WG. The couple agreed to an external examination of the fetus only. The weight of the male fetus was evaluated at the 60th percentile (1295 g) as well as the length (crown-heel: 41 cm) and head circumference (28 cm). External examination revealed a craniofacial dysmorphism including dolichocephaly, hypertelorism, epicanthus, proptosis, convex nasal ridge, retrognathia, micrognathia, and small and low-set ears with prominent antitragus, underfolded helix, and absence of right earlobe. In addition, bilateral 2-3 toes' cutaneous syndactyly, extreme flexion contracture of the hallux, and talipes equinovarus were associated (Figure 1(a)). Skeletal X-ray and placental examination were normal according to gestational age.

## 3. Cytogenetics

Chromosomes were obtained using conventional cytogenetic techniques from amniotic fluid cells. Standard karyotype analysis performed on cultured amniotic fluid cells showed a normal male 46,XY karyotype. Agilent oligonucleotide Human PreCytoNem 105K array-CGH (Agilent Technologies, Santa Clara, California, USA) was used to detect chromosomal abnormalities in the fetus. Array-CGH realized on DNA obtained from uncultured amniotic fluid cells revealed an interstitial deletion of the long arm of chromosome 17 (Figure 1(b)). The proximal breakpoint was located on 17q24.1 (position min 63,739,282, position max 63,685,334) and the distal breakpoint was located on 17q24.2 (position min 66,303,332, position max 66,364,849) (genome build hg19). Thus a minimal 2.5 Mb region was deleted. A total of 20 genes are referenced within the deleted region (Supplementary Table I in Supplementary Material available online at https://doi.org/10.1155/2017/7803136). Array-CGH results were confirmed by FISH (Fluorescence In Situ Hybridization) using BAC (Bacterial Artificial Chromosome) probes. FISH analyses were performed on metaphase spreads and interphasic nuclei of both cultured amniotic fluid cells and parental lymphocytes. BAC clones specific for the 17q chromosomal regions were used (RP11-74H8 and RP11-162L11 located at 17q24.2, RP11-342F21 located at 17q12) (Bluegnome, Amplitech, Compiègne, France). Both RP11-74H8 and RP11-162L11 BAC probes gave one signal on normal chromosome 17 and no signal on deleted chromosome 17 (data not shown). FISH analyses on parents' cells were normal and thus indicated a de novo origin for the deletion. In summary, the fetus had a 2.5 Mb de novo 17q24.1q24.2 deletion. Based on the ISCN 2016 nomenclature, the formula was as follows:

  46,XY.ish del(17)(q24.2q24.2)(RP11-74H8-,RP11-162L11-)dn
  .arr[hg19] 17q24.1q24.2(63739282_66303332)x1 dn

## 4. Discussion

Deletion of the 17q24.1q24.2 region is an entity described postnatally in patients suffering from intellectual disability, abnormality of the facial shape, and/or malformations. To our knowledge this is the first report in a fetus. Among the previously described patients, 20 patients were analyzed using array-CGH. Among them, 13 cases of interstitial deletion of 17q region overlapping with the deletion of the fetus were phenotypically described (Table 1). Eleven cases were published and 2 were described in DECIPHER database [9] (Figure 1(c)). Most of these patients presented mental or psychomotor delays in association with abnormality of the fingers or toes (2-3 toes' syndactyly), obesity, failure to thrive, and facial dysmorphism with hypertelorism, prominent nose, ears abnormality (malformation or low-set ears), thin lips, and teeth abnormality (Table 1). We observed some common features with the patient described by Blyth et al.'s study presenting hypertelorism, bilateral 2-3 toes' syndactyly, beaking of the nose, and epicanthus [4]. The deletion 17q24.2q24.3 was larger than ours and other features were absent or nonevaluable in prenatal examination (postnatal growth retardation, freckles, and lentigines). Vergult et al. described a new 17q24.2 microdeletion syndrome characterized by the association of intellectual disability, pronounced speech delay, truncal obesity, and facial dysmorphism [5]. After this first description, two other cases were published with the same smallest region of overlap and phenotype close to cases of Vergult et al.'s study [6, 7]. More recently, Küry et al. published a cohort of patients with mutations or CNV deletions encompassing* PSMD12*, located in 17q24.2 [8]. These patients presented intellectual disability associated with craniofacial dysmorphism. We identified 20 known coding genes within the deleted region of our case (Supplementary Table I). Among them, 14 genes are OMIM morbid genes. Some of them were associated with human pathogenicity. Among them we discuss* KPNA2 *and* PSMD12 *genes, also deleted in the patients of Stewart, Bartnik, Vergult (patients 3 and 4), and Küry (patients 5 and 6) studies [5–8].* KPNA2 *encodes for karyopherin alpha-2 and is involved in the nuclear import of proteins.* KPNA2* is involved in the Nijmegen breakage syndrome (NBS). KPNA2 interacts with the NBS gene product NBS1 involved in checkpoint arrest and repair-response to DNA double-strand breaks [10]. Mutations in* NBS1* were identified in most patients with NBS [10]. The NBS is a rare autosomal recessive syndrome characterized by microcephaly at birth, intrauterine growth retardation, short stature, chromosomal instability, immunodeficiency, and predisposition to malignancy [11]. Craniofacial features can associate palpable anterior fontanel, prominent midface, sloping forehead, retrognathia, upslanted palpebral fissures, long, beaked, or upturned with anteverted nostrils nose, and in half of the patients clinodactyly of the 5th fingers and partial 2-3 toes' syndactyly [11]. Our case presented some common characteristics with patients encompassing NBS: retrognathia, convex nasal ridge, and cutaneous 2-3 toes' syndactyly. The proposed mechanism in this case is* KPNA2* haploinsufficiency responsible for decreased expression of KPNA2.* KPNA2* has a high haploinsufficiency score in DECIPHER database (HI index = 10.31%), and it is more likely to exhibit haploinsufficiency [12]. Moreover, according to the ExAC browser, with a probability of loss-of-function intolerance of 0.73,* KPNA2* is predicted to be moderately intolerant to loss-of-function mutations [13].* PSMD12* encodes the subunit PSMD12 of the 26S proteasome, involved in degradation of polyubiquitinated proteins. Utilizing zebrafish, Küry et al. revealed that* PSMD12* was important in brain, renal, and craniofacial development [8]. Thereby,* PSMD12* haploinsufficiency could explain craniofacial dysmorphism observed in our case. The mother of the fetus had in her clinical history clubfeet at birth, without chromosomal rearrangement. With this information, it is difficult to establish a genotype-phenotype correlation between the 17q24.1q24.2 deletion and the deformities of the feet observed in the fetus.

In conclusion we reported the first case of prenatal diagnosis of 17q24.1q24.2 deletion in a fetus with polyhydramnios, abnormal facial shape, 2-3 toes' syndactyly, and talipes equinovarus. The deletion encompassed* KPNA2 *and* PSMD12 *which could be involved in the fetal phenotype associated with 17q24.1q24.2 deletion. This report highlights the necessity to perform array-CGH also in prenatal diagnosis in case of minor echographic signs association.

## Supplementary Material

Supplementary Table I: The description and the physiological role of the genes included in the 17q24.1q24.2 deleted region are presented with the OMIM reference. The impact of their dysregulation is detailed when described in animal models and in human species.

## Figures and Tables

**Table 1:** Phenotypes of the patients described by array-CGH with a deletion overlapping with our case.

	Decipher 300694	Decipher 273548	Blyth et al. 2008 [4]	Vergult et al. 2012 [5] Patient 1	Vergult et al. 2012 [5] Patient 2	Vergult et al. 2012 [5] Patient 3	Vergult et al. 2012 [5] Patient 4	Bartnik et al. 2014 [6]	Stewart et al. 2014 [7]	Küry et al. 2017 [8] Patient 5	Küry et al. 2017 [8] Patient 6	Küry et al. 2017 [8] Patient 9	Küry et al. 2017 [8] Patient 10	Present Case
17q deletion (cytoband)	q24.2	q24.2	q24.2q24.3	q23.3q24.2	q24.1q24.2	q24.2	q24.2q24.3	q24.2	q24.2q24.3	q23.3q24.2	q24.2	q24.2	q24.2	q24.1q24.2
Size (Mb)	1,19	0,23	2,26	3,11	1,71	2,11	4,16	1,9	3,89	1,37	4,06	0,84	0,62	2,5
Age at phenotypic description	Childhood	Postnatal	12 years	28 years	11 years	28 years	2.5 years	1 year	16 years	1.5 years	3.5 years	4.5 years	9 years	Prenatal
Parental origin	DN	MH	DN	DN	DN	DN	DN	DN	DN	DN	DN	DN	DN	DN
Craniofacial dysmorphism	+	+	+	+	+	+	+	+	+	+	+	+	+	+
Triangular face	−	−	−	−	+	−	+	NR	−	−	−	−	−	−
Round face	−	−	−	−	+	+	+	NR	−	−	−	−	−	−
Small/narrow palpebral fissures	−	−	−	−	−	+	+	NR	+	−	−	−	−	−
Downlanting palpebral fissures	+	−	−	+	−	−	−	NR	−	−	−	−	−	−
Ptosis	−	−	−	−	+	+	+	NR	−	−	−	−	−	NA
Hypertelorism	+	−	+	+	+	+	+	NR	+	+	+	−	−	+
Bulbous/prominent nose	−	−	+	−	+	+	+	NR	+	−	−	+	+	−
Broad nasal bridge	−	−	+	−	−	+	+	NR	+	−	−	−	−	−
Short philtrum	−	−	−	−	−	+	−	NR	+	−	−	+	−	−
Thin lips	+	−	+	+	+	+	+	NR	NR	−	−	−	−	−
Arched or cleft palate	−	−	+	−	+	−	−	NR	−	−	−	+	−	−
Abnormality of the teeth	−	−	+	+	−	+	+	NR	+	−	−	−	−	NA
Retrognathia/micrognathia	−	−	+	−	+	−	+	NR	−	+	−	+	−	+
Ears abnormality	−	−	−	+	+	+	+	NR	+	−	+	+	+	+
Dolichocephaly	−	−	−	NR	NR	NR	NR	NR	−	−	−	−	−	+
Microcephaly	−	+	+	−	−	−	+	NR	−	+	−	−	−	−
Hearing impairment	−	−	−	−	+	+	+	NR	−	−	+	−	−	NA
Visual impairment	−	−	+	+	−	−	−	NR	+	−	−	−	−	NA
Malformation of the heart/great vessels	−	−	NR	+	−	NR	+	NR	+	+	+	−	−	NA
Mental/psychomotor retardation	+	+	+	+	+	+	+	+	+	+	+	+	+	NA
Behavioural/psychiatric abnormality	−	−	−	+	−	+	−	NR	+	−	NR	NR	+	NA
Seizures	−	−	−	+	−	+	−	NR	−	−	−	−	+	NA
Polyhydramnios	−	−	NR	−	NR	NR	NR	NR	−	NR	NR	NR	−	+
Intrauterine growth retardation	−	−	+	−	−	+	+	NR	+	NR	+	+	+	−
Feeding difficulties	−	−	+	+	+	+	+	NR	+	−	+	+	−	NA
Failure to thrive/short stature	+	−	+	−	+	+	+	NR	+	+	+	+	−	NA
Obesity	−	−	−	+	+	+	+	+	−	−	−	−	−	NA
Skeletal features	−	−	NR	+	+	+	NR	NR	+	−	−	+	−	−
Joint anomalies	−	−	NR	+	−	+	+	NR	−	−	−	−	−	+
Abnormality of skin	−	−	+	−	−	+	+	NR	+	−	−	−	−	−
Equinovarus feet	−	−	NR	−	−	−	NR	NR	−	−	−	−	−	+
Abnormality of the fingers/toes	−	−	+	+	+	+	+	NR	+	−	+	+	−	+
Clinodactyly of fifth finger/toes	−	−	+	−	+	+	−	NR	−	−	−	−	−	−
2-3 toes' syndactyly	−	−	+	−	−	+	+	NR	+	−	+	+	−	+

+: clinical feature present; −: clinical feature absent; NA: not applicable; NR: not reported; DN: de novo; MH: mother inherited.

## Additional Points

*Web Resources.* This study makes use of data generated by the DECIPHER community. A full list of centres who contributed to the generation of the data is available from https://decipher.sanger.ac.uk and via email from decipher@sanger.ac.uk.
