# Supplementary material for: Prenatal Diagnosis of a 2.5 Mb De Novo 17q24.1q24.2 Deletion Encompassing KPNA2 and PSMD12 Genes in a Fetus with Craniofacial Dysmorphism, Equinovarus Feet, and Syndactyly
Source: Case Rep Genet. 2017 Mar 29;2017:7803136. doi: 10.1155/2017/7803136 (PMC5390532; doi:10.1155/2017/7803136)
Supplement: Supplementary file 1 — Supplementary Table I: The description and the physiological role of the genes included in the 17q24.1q24.2 deleted region are presented with the OMIM reference. The impact of their dysregulation is detailed when described in animal models and in human species. [file 7803136.f1.docx]

Supplementary table I. Description of the genes mapped in the deleted region.

| **Gene** | **Description** | **OMIM n°** | **Function** | **Animal model (species)** | **Human pathogenicity** |
| --- | --- | --- | --- | --- | --- |
| ***CEP112*** | centrosomal protein 112kDa | **/** | Cell division control |  |  |
| ***APOH*** | apolipoprotein H (beta-2-glycoprotein I) | **138700** | Lipoprotein metabolism, coagulation, hemostasis, production of antiphospholipid autoantibodies and apoptosis | Decrease of implantation and thrombin generation (mice) |  |
| ***PRKCA*** | protein kinase C, alpha | **176960** | Diverse cellular processes (cell adhesion, cell transformation, cell cycle checkpoint, cell volume control), serve as receptor for phorbol esters | Cardiac hypercontractility (mice) | Altered BMI, asthma, schizophrenia, candidate for intellectual disability |
| ***MIR634*** | microRNA 634 | **/** | Post-transcriptional regulation of gene expression |  |  |
| ***CACNG5*** | calcium channel, voltage-dependent, gamma subunit 5 | **606405** | Trafficking and channel gating of AMPA- selective glutamate receptors (AMPARs) |  | Susceptibility locus for schizophrenia and bipolar disorder |
| ***CACNG4*** | calcium channel, voltage-dependent, gamma subunit 4 | **606404** | Trafficking and channel gating of AMPA- selective glutamate receptors (AMPARs) | Seizure when *CACNG2* is also disturbed (mice) | Susceptibility locus for schizophrenia and bipolar disorder |
| ***CACNG1*** | calcium channel, voltage-dependent, gamma subunit 1 | **114209** | Excitation-contraction coupling |  | Susceptibility locus for schizophrenia and bipolar disorder |
| ***HELZ*** | helicase with zinc finger | **606699** | Conformation of RNA (biologic activity and access to other proteins) | Role in RNA metabolism in tissues and organs within the developing embryo (mice) |  |
| ***PSMD12*** | proteasome (prosome, macropain) 26S subunit, non-ATPase, 12 | **604450** | ATP-dependent degradation of a variety of cellular proteins | Role in development of optic tecta, proximal tubule convolution and craniofacial cartilage in zebrafish embryo |  |
| ***PITPNC1*** | phosphatidylinositol transfer protein, cytoplasmic 1 | **605134** | Multiple processes including cell signaling and lipid metabolism by facilitating the transfer of phosphatidylinositol between membrane compartments |  |  |
| ***NOL11*** | nucleolar protein 11 | **615366** | Component of the ribosomal small subunit processing subcomplex, required for pre-rRNA transcription and processing | Reduce pre-rRNA transcription by polymerase I (HeLa cells) |  |
| ***SNORA38B*** | small nucleolar RNA, H/ACA box 38B | **/** |  |  |  |
| ***BPTF*** | bromodomain PHD finger transcription factor | **601819** | Regulation of transcription | Inhibits osteoblast differentiation, compromises spatial control of Hox gene expression (Xenopus embryos) |  |
| ***C17orf58*** | chromosome 17 open reading frame 58 | **/** |  |  |  |
| ***KPNA2*** | karyopherin alpha 2 (RAG cohort 1, importin alpha 1) | **600685** | Nuclear transport of proteins and V(D)J recombination |  | Candidate for Russel-Silver Syndrome, associated with Nijmegen breakage syndrome |
| ***LINC00674*** | long intergenic non-protein coding RNA 674 | **/** |  |  |  |
| ***LOC440461*** | Rho GTPase activating protein 27 pseudogene | **/** |  |  |  |
| ***AMZ2*** | archaelysin family metallopeptidase 2 | **615169** | Zinc metalloprotease |  |  |
| ***ARSG*** | arylsulfatase G | **610008** | Hormone biosynthesis, modulation of cell signaling, degradation of macromolecules, arylsulfatase activity at acidic pH with pseudosubstrates | Adult-onset cerebellar ataxia (dogs) | Candidate for human adult-onset cerebellar ataxia |
| ***SLC16A6*** | solute carrier family 16, member 6 | **603880** | Member of the proton-linked monocarboxylate transporter (MCT) family: catalyzes Lactic acid and transports pyruvate across plasma membranes |  |  |
